# Comprehensive Evaluation of Late Season Peach Varieties (*Prunus persica* L.): Fruit Nutritional Quality and Phytochemicals

**DOI:** 10.3390/molecules26092818

**Published:** 2021-05-10

**Authors:** Dasha Mihaylova, Aneta Popova, Ivelina Desseva, Ivan Manolov, Nadezhda Petkova, Radka Vrancheva, Alexander Peltekov, Anton Slavov, Argir Zhivondov

**Affiliations:** 1 Department of Biotechnology, Technological Faculty, University of Food Technologies, 4002 Plovdiv, Bulgaria; 2 Department of Catering and Tourism, Economics Faculty, University of Food Technologies, 4002 Plovdiv, Bulgaria; popova_aneta@yahoo.com; 3 Department of Analytical Chemistry and Physical Chemistry, Technological Faculty, University of Food Technologies, 4002 Plovdiv, Bulgaria; radka_vrancheva@yahoo.com; 4 Department of Agrochemistry and Soil Science, Agricultural University, 4000 Plovdiv, Bulgaria; manolov_ig@yahoo.com; 5 Department of Organic Chemistry and Inorganic Chemistry, Technological Faculty, University of Food Technologies, 4002 Plovdiv, Bulgaria; petkovanadejda@abv.bg (N.P.); antons@uni-plovdiv.net (A.S.); 6 Accredited Laboratory Complex for Chemical and Instrumental Analysis, Agricultural University, 4000 Plovdiv, Bulgaria; alexander_peltekov@abv.bg; 7 Fruit Growing Institute, 4000 Plovdiv, Bulgaria; a.zhivondov@abv.bg

**Keywords:** peach, soil nutrients, phytochemical composition, antioxidant capacity

## Abstract

Peaches are one of the most preferred seasonal fruits, and a reliable source of nutrients. They possess biologically active substances that largely differ among varieties. Hence, revealing the potential of several late season peaches is of present interest. Three commonly consumed varieties (“Flat Queen”; “Evmolpiya”; “Morsiani 90”) were studied in terms of nutritive and phytochemical content, as well as antioxidant activity with the use of reliable spectrophotometric and High Performance Liquid Chromatographic (HPLC) methods. An analysis of the soil was also made. The phytochemical data were subjected to principal component analysis in order to evaluate their relationship. The “Morsiani 90” variety had the highest minerals concentration (2349.03 mg/kg fw), total carbohydrates (16.21 g/100 g fw), and α-tocopherol (395.75 µg/100 g fresh weight (fw)). Similar amounts of TDF (approx. 3 g/100 g fw) were reported for all three varieties. “Flat Queen’s” peel extract was the richest in monomeric anthocyanins (2279.33 µg cyanidin-3-glucoside (C3GE)/100 g fw). The “Morsiani 90” variety extracts had the highest antioxidant potential, defined by 2,2-diphenil-1-picrylhydrazyl (DPPH), ferric-reducing antioxidant power (FRAP) and cupric ion-reducing antioxidant capacity (CUPRAC) assays.

## 1. Introduction

Research shows that fruit intake is still inadequate in many countries and health-promoting strategies are much needed among the population [1]. Fruits are valuable sources of nutrients and their consumption is associated with well-established positive physical effects, as well as psychosocial ones [2]. Many factors are involved in fruits and vegetable quality including genotypic, agro-environmental and postharvest ones [3,4,5]. However, the nutritional value is not sufficient. Consumers most often prefer products that possess the so-called biologically active substances (BAS), i.e., different types of compounds (dietary fiber, phenolic compounds, carotenoids, vitamins etc.) that have impact on the human body’s functions. Among them, polyphenols are, perhaps, the most recognizable and sought-after compounds. They possess antioxidant, antimicrobial and anti-cariogenic properties [6,7]. The distribution of BAS in fruit is not homogeneous. That is why, the consumption of the whole fruit is recommended. There is increasing evidence that consumption of whole foods is better than isolated food components, such as dietary supplements and nutraceuticals [8]. This is due to the food matrix effect that influences the behavior of individual components [9]. Thus, for instance, the combination of different fruits and vegetables leads to an additive and synergistic effect of their individual antioxidant and antiproliferative activities [10,11]. 

**Figure 1:**
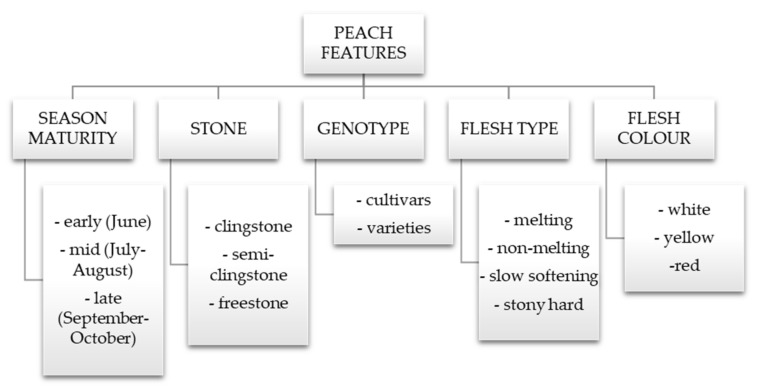
Peach features [14,15].

Agricultural produce will always interest different target audiences (researchers, agronomists, chemists, food specialists, etc.), and over the past years, the agro-food sector is turning to sustainability by paying close attention to food quality, waste, resources, etc. [12]. The numerous distinct peach varieties possess different quality features, i.e., fruit yield, spring frost damage adaptability, fruit size and color, chemical, and sensory attributes. Peach varieties are yearly introduced to different regions worldwide, making the peach (Prunus persica L.) one of the main fruit trees ensuring fresh tasty fruits during the long summer period—from June to October. The peach flavor is very dependent on the chemical composition, storage duration and conditions [13]. Peaches can be classified according to many characteristics: season availability, flesh featuring, stone attribute, etc. (Figure 1).

Peaches contain minerals, sugars, organic acids, dietary fiber, vitamins [16]. This rich chemical palette is associated with many important biochemical transformations in the human body [17]. Peach dietary fiber is a valuable nutritious component in both, peel and pulp [18], that plays a beneficial role in the gastrointestinal health. Moreover, peaches possess compounds with antioxidant properties—ascorbic acid, carotenoids [19], phenolic acids, flavonoids, and anthocyanin [20,21,22]. However, large content variations exist between varieties. Research shows that a variety being local or introduced can exhibit unique characteristics, and it is always challenging to evaluate the performance of these varieties. In this regard, every comprehensive research can add up to the important consumer-oriented database for each variety. 

Thus, the present work aims at investigating and comparing key physical, biochemical, nutritional, and phytochemical aspects of three late season peach varieties—“Flat Queen”; “Evmolpiya”; “Morsiani 90”, grown in Bulgaria. Several spectrophotometric and HPLC methods have been applied in order to evaluate the main compounds of the samples. In addition, soil analyzes were also assessed. To ensure a complete featuring, four methods for antioxidant evaluation, including DPPH (1,1-diphenyl-2-picrylhydrazyl) assay, ABTS (2,2′-azinobis-(3-ethylbenzothiazoline-6-sulfonic acid) assay, FRAP (ferric reducing antioxidant power) assay and CUPRAC (cupric reducing antioxidant capacity) assay were used in this study. Furthermore, principal component analysis (PCA) was applied to confirm extracts’ differences or similarities.

## 2. Results and Discussion

Table 1 shows a visual presentation of the soil characteristics, studied in the current investigation. Soil reaction (pH) strongly affects plant growth and development [23]. Although, it is the most routinely measured parameter, it plays a significant role in many soil processes [24]. In the present study, the three researched varieties were grown in the same orchard, so aliquots were taken twice before harvest—in March and in July. 

The main part of the root system of fruit species develops in the one-meter soil layer, even though individual roots can reach a depth of several meters. Samples from three depths were collected and analyzed (0–25 cm; 25–50 cm; 50–75 cm). The pH was meas-ured in two extracts—aqueous and saline (1 M KCl). A soil suspension ratio of 1:5 was prepared. The soil reaction was slightly alkaline when measured in a water extract, and neutral in a potassium chloride extract. This difference is expected, due to the displace-ment and release of protons in the case of KCl compared to distilled water [24]. However, in July 2020, the soil pH profile was slightly alkaline at all studied depths for both ex-tracts.

The salt concentration of the soil (EC) was very low. On the first sampling day, it ranged from 63.90 to 76.30 µS/cm, while on the second, it was 100.40–145.60 µS/cm. The carbonates content was less than 1.5 g/kg soil in all measurements. The soil during the beginning of the peach vegetation (2 March 2020) can be characterized as well stocked with mineral nitrogen (Table 1), especially with the weakly mobile ammonium form of the element. Its content varied from 214.54 to 239.52 mg/kg on the first sampling date, and dropped to 4.38–7.07 mg/kg in July. Only in the upper 0–25 cm horizon, 285.12 mg/kg of nitrates were found. This can be explained by the earlier warming of this horizon and the beginning of the microbiological nitrification process. Less than 0.50 mg/kg were measured, in all other horizons. The concentration of absorbable forms of nitrogen in the soil decreased sharply between sampling. 

Needs of phosphorus, for most fruit crops, are relatively low [25]. That is why in-dependently of comparatively low content of available phosphorus in soil peach trees can satisfy their needs of this nutrient relying on their deep and well-developed root system. In this study, moderate phosphorus concertation was detected in the two upper layers of the soil, ranging from 5.21 to 8.44 mg P_2_O_5_/100 g in March. During the summer, the amount of mobile phosphorus has decreased (2.56 to 4.47 mg P_2_O_5_/100 g), and it can be classified as low in the three soil layers. This is due to the fact, that the garden was not fertilized with phosphorus and potassium fertilizers at all. 

Available K, exchangeable Ca, and exchangeable Mg were analyzed as well; increase of the concentrations between sampling was noted. Moreover, Bulgarian soils generally have large reserves of potassium. The content of available potassium into the soil depends on soil texture. It is higher in fine-textured soils [25], what is the soil in the peach plantation. The content of sulfates decreases in depth and between sampling of the soil profile, but the amounts were sufficient for the normal development of the trees. 

The content of boron and molybdenum was very low—below 1.00 mg/kg (data not presented). The heavy metals cadmium, cobalt and nickel were practically absent in the studied soil (below <1.00 mg/kg—data not presented). The lead content was far below the maximum permissible concentration of lead in soil with pH_H_2_O_ > 7.4 (100 mg/kg) of soil according to the Bulgarian state standard [26]. The chromium content was also very low, with no clear tendency to change the depth of the soil profile. 

The clay content was the highest in the upper soil layer 47.75%, which defines the soil as heavy sandy clay according to Kaczynski’s classification [27]. The amount of clay in the lower horizons decreased to 37.50% in the 25–50 cm layer and 31.25% in the 50–75 cm layer. The soil in these two layers is defined as slightly sandy loam. Most soil nitrogen accumulates in soil organic matter. The presence of humus in the soil will release about 10 kg of nitrogen per year per ha [25]. According to the humus content in the top two layers, the soil was characterized as poor in humus (2.61 and 2.91%, respectively). The lowest layer was even poorer (0.74%). 

Nitrogen is reported to have a directly proportional influence of TSS values [28], which, in the current study, does not increase with season progression and prolonged N availability. Nitrogen can also greatly affect the phenolic content of peaches, where the lowering of N values leads to an increased phenolic content, in most cases. 

### 2.1. Fruit Characteristics

Different physicochemical parameters were evaluated, i.e., fruit and pit weight, firmness, content of soluble solids, dry matter, ash, pH and titratable acidity, in the pre-sent study (Table 2). The average fruit weight ranged from 185.63 to 221.88 g, wherein the heaviest fruits were those of the “Evmolpiya” variety. The fruit weight was considered as highly dependent on the harvest time, cultivar, fruit load, and on climatic and agricultural conditions [29,30]. In general, the later ripening peach varieties had heavier fruits than those harvested earlier and vice versa [31], which is in agreement with the currently established. The lightest pit was weighted for “Flat Queen”. The stones of “Evmolpiya” and “Morsiani 90” did not differ statistically. In this regard, the stone mass was not in a clear relationship with the fruit weight, and could be assumed as specific for the variety, as already reported by Nowicka et al. [17]. 

Firmness and fruit quality are strongly linked to each other. Firmness can be affected by cell wall strength [32]. Regarding the firmness and dry matter, “Morsiani 90” dis-played the highest values—5.75 kg/cm^2^ and 20.72%. Higher firmness means greater transport stability and longer life after harvest [33]. The values were a little bit higher than the results reported by Aubert and Chalot [34] who found a variation of dry matter content in 14 cultivars in the range from 10.5 to 18.0%. The ash content is reported as dependent on the growing conditions, in addition to the variety itself, which includes climatic influences [35]. In this study, it varied from 0.68 to 1.36% for “Evmolpiya” and “Morsiani 90. The total soluble solids were 14.44 (“Flat Queen”), 14.50 (“Evmolpiya”), and 13.54% (“Morsiani 90”). The TSS content in peach fruits was higher compared to the results reported by other researchers [34]. Acid pH was measured for all varieties; the lowest was detected in “Morsiani 90”—3.51.

Varieties with the same flesh firmness like “Flat Queen” and “Evmolpiya” showed different pH and TA values, but similar TSS, which implies that not only the firmness, but also other chemical attributes should be taken into account to the maturity stage. The maturity index (MI) of the fruits was analyzed in this study (Table 2) as an indicator of the relationship between total soluble solids content and acids, which is an important index of fruit flavor [17]. The maturity index of “Flat Queen” was calculated to be 32.09, while other two varieties had 1/3 lower indices (22.31 and 23.34).

Burdon et al. [36] suggests that dry matter can be a maturity indicator and sensory quality predictor, as it includes both soluble and insoluble solids. Results obtained in this study partly disagree with this theory since “Morsiani 90”, the variety with the highest dry matter content, did not show a higher TSS value, although it contained the most total carbohydrates.

Color assessment is an important analysis that could predict fruit maturity and ca-rotenoid content [37,38]. The use of a colorimeter in order to determine the CIELAB color attributes, guarantees objective measurements and better determination [39,40]. Table 3 is a visual presentation of the CIELAB skin and flesh color measurements data of the studied peach varieties. No statistically significant variations of the skin and flesh lightness was reported between varieties. The hue angle values corresponded to the reddish-orange color for the skin, and the yellow color for the flesh. The chroma (color saturation) varied significantly among samples (for both skin and flesh). 

Based on the CIELAB results, the total color difference was calculated and the three varieties were compared to each other. ΔEskin of “Flat Queen” compared to “Evmolpiya”, and “Morsiani 90” was 19.11 and 10.44, resp. ΔEskin of the comparison between “Evmolpiya” and “Morsiani 90” resulted in a value of 25.14. ΔEflesh for the same pair of varieties was 34.86, 28.41 and 7.44, resp.

Flesh color aids in the explanation of some quality and nutritional variations in peaches and nectarines. It is usually associated with maturity, phenolic acid and carotenoid contents. Values of “a” and hue flesh parameters can be used to determine the maturity of yellow fleshed peaches. Color measurement is an excellent non-destructive way of ripeness prediction.

### 2.2. Mineral Composition

The mineral composition was assessed as well, and the results are presented in Table 4. The total mineral content of the varieties varied from 683.42 to 2349.03 mg/kg fw. Potassium (K) is the main macronutrient found in peaches [22,41]. The potassium has positive effect on human health. The increased intake of this nutrient lowers blood pressure, reduces cardiovascular disease mortality, and prevents the progression of renal disease [42]. Although the soil contains sufficient amounts of available potassium (Table 1), the three varieties included in the study accumulated quite different amounts of the nutrient in the fruits. “Morsiani 90” showed the highest content of K (1978.34, mg/kg fw), followed by “Evmolpiya” (738.48 mg/kg fw). The content of potassium in the variety “Flat Queen” was 5.6 times lower compared to “Morsiani 90” (353.37 mg/kg fw). Manzoor at al. [22] did not find considerable differences in the fruits of three peach varieties grown in Pakistan. The content of P was the same in the fruits of “Flat Queen” and “Morsiani 90” (300 mg/kg fw). The content of this element in “Evmolpiya” was 200 mg/kg fw. The highest K and Na content was found in “Morsiani 90” (1978.34, 300.00 and 55.92 mg/kg fw), while the lowest was found in “Flat Queen” (353.37 and 24.57 mg/kg body weight). Fe and Zn, as essential microelements, are unevenly distributed among the varieties, in the range from 0.44–0.62 to 0.32–1.18 mg/kg fw. Iron was the most abundant element in “Evmolpiya”, while Zn—in “Morsiani 90”. Both elements are important. Iron as an essential trace element and a core of red blood cells, a deficiency of which can cause anemia [43], and zinc—as a coenzyme for over 200 enzymes involved in immunity, new cell growth, acid base regulation etc. [44]. The analyzed varieties contained very small amounts of Ca, less than 0.13 mg Ca/kg fw. The content of magnesium was the highest in the nectarine variety “Morsiani 90” (11.84 mg mg/kg fw). The content of this element was almost 3 times lower in “Flat Queen” and 4 times lower in “Evmolpiya”. The soil contented considerable amounts of exchangeable Mg, which can be absorbed by plants; even this content was increased in the summer, i.e., near the maturity of fruits (Table 1). Therefore, the differences in magnesium content are mainly due to variety peculiarity. The highest content of Mn (0.22 mg/kg fw) and Cu (0.89 mg/kg fw) was quantified in “Morsiani 90”. Pb and Cr were found in less than 0.10 mg/kg fw, in all studied varieties. “Flat Queen” displayed significantly higher content of nitrogen—9.80 g/kg fw than “Evmolpiya” (1.0 g/kg fw) and “Morsiani 90” (4.1 g/kg fw). 

A similar element profile has been reported for numerous peach varieties [41,45,46]. However, a variety-specific composition should be taken into account [45]. Potassium intake has been positively associated with bone metabolism, lower blood pressure and reduced morbidity and mortality from cardiovascular disease [46,47]. Zinc, copper and manganese, on the other hand, are essential microelements for human enzymes metabo-lism [48]. Manganese is one of the important essential elements, but it is required only in trace amounts to maintain proper carbohydrates metabolism, as well as an antioxidant in superoxide dismutase enzymes [43]. Magnesium plays an important role in the stability of the nervous system, muscle contraction, as an activator of alkaline phosphatase, and can also be used as an alternative to calcium in the body [49].

The Kjeldahl method was used to assess the nitrogen content. Values ranges from 1.0 to 9.8 g/kg fw, including small amounts of protein nitrogen.

### 2.3. Nutrient and Phytochemical Characteristics

Carbohydrates (especially fructose, sorbitol and glucose), lipids and vitamins, among others, are considered basic fruit components [50]. Table 5 presents the results for the carbohydrates, total lipids, dietary fiber, carotenoids, and tocopherols of the late ripening peach varieties, as well as their calculated energy equivalence. Sucrose was the major sugar in all varieties, and its value was the highest in “Flat Queen”—4.21 g/100 g fw. According to Cirilli et al. [51] sucrose is the predominant sugar in the peach mesocarp at maturity, accounting for approximately 40–85% of the total sugars content. Glucose and fructose (in variable ratios), represent approximately 10–25%, and sorbitol accounts for less than 10%. In the current case, the lowest level of sorbitol was detected in “Evmolpiya” (0.09 g/100 g fw), while the highest—in “Morsiani 90” (0.47 g/100 g fw), accounting for approximately 10% of total sugars. A small amount of fructose, and large amount of sorbitol were found in peaches suggesting the existence of an inhibitor or a weak enzyme activity in the metabolic process into fructose [52]. Sorbitol is a typical translocating substance in all representatives of the Rosacea family (apples and peaches), that can be converted to fructose or glucose by NAD+-dependent sorbitol dehydrogenase and sorbitol oxidase, respectively [51]. Fruits with low fructose:glucose ratio are reported to have higher fructokinase and lower neutral invertase capacities [13], which is the case of the “Morsiani 90” variety with a ratio of 0.18. This variety should also have a lower ability to catalyze sucrose into fructose and glucose.

Glucose was the second sugar, in high amounts, in the investigated samples. Glucose was the most abundant in the “Evmolpiya” peaches (1.60/100 g fw), which were also rich in fructose (0.95/100 g fw), and together with glucose brought the highest value of reducing sugars in this variety (6.19/100 g fw). Contrary to the statement of Nowicka et al. [17], the glucose content was higher than that of fructose in all three peach varieties. Robertson and Meredith [53] suggested that low-quality peaches contain lower fructose and higher sorbitol and glucose compared to high-quality peaches.

The total sugar value varies from 4.97 to 6.19/100 g fw, which coincides well with the results reported by other investigators (4.6–9.6%) [41,54]. Ninety-four traditional Spanish (mostly non-melting flesh types) varieties had similar values, where the total sugar con-tent varied from 63 to 136 g/kg FW (Suc 35–98, Glc 4–15, Fru 2–14, and Sor 2–35) [55]. The highest values of total carbohydrates were found in the “Morsiani 90” peach—16 g/100 g fw. More than 55% of the total carbohydrates content in the “Flat Queen” variety was due to the reducing sugars, and below 30% was occupied by sucrose, as dominating sugar. The sucrose/glucose ratio in all three varieties was above 2.3 and reached a maximum of 5.47 for “Flat Queen”. This fact clearly indicated that sucrose is mainly responsible for the sweetness of these peaches. Sugars also affect mouth-feel attributes and aroma perception. Sucrose and sorbitol are highly correlated with the overall taste and aroma of fruits [51]. For all three peach varieties, the glucose/fructose ratio was 1.8, which coincides with a previous report for peach varieties grown in Bulgaria [41].

The perception of taste may be affected by many factors. Several indices were used to characterize peach fruit quality. Sweetness (SI) and total sweetness indices (TSI) are good indicators of the content of sucrose, glucose and fructose in the fruit (Table 5). Each carbohydrate contributes differently in the calculation of the index with fructose having the highest coefficient. The variety with the highest TSS, has the highest SI and TSI as well, which corresponds to the carbohydrate content of the fruit, where glucose and fructose are predominant. All of the studied varieties can be considered sweet with a ratio of 1.1:1.3:1.0 (“Flat Queen”: “Evmolpiya”: “Morsiani 90”) corresponding to their size and TSS values. The “Evmolpiya” variety demonstrated the highest value of the SI (87.0) and TSI (62.8), while the lowest values were observed in the “Morsiani 90” variety (64.8 and 47.7, respectively). The sweetness intensity depends on the overall sugar amount, as well as on the specific sugar profile. However, the total acidity strongly affects the sweetness perception. Sweetness is mainly correlated with the sugars:acids ratio, and the overall organic acid concentration [56,57]. The soluble sugars and acid ratio determines the maturity and taste of the peach. The total sugars/total titratable acidity ratio of the three peach varieties “Flat Queen”, “Evmolpiya”, “Morsiani 90” varied from 12 to 21. In fruits, including peaches, this ratio is used to classify them as sour (MI: 5–7), sour–sweet (MI: 17–24), and sweet (MI: 31–98) [58]. It can be concluded that the “Evmolpiya” variety was sour-sweet, while the other two varieties were semi-sweet. The currently established data coincided well with the values documented by other authors for European peach cultivars (13.24 (‘SB6A–35′peach) to 26.59 (“Royalvee “ peach)) [17].

The lipid content in the analyzed peaches ranged from 0.61 g/100 fw to 0.46 g/100 g fw. The highest lipid content was detected in the “Evmolpiya” variety, which coincided with previously reported data for mid-ripening peach varieties [41]. The total lipids in the cv. Redhaven peach [59] were closer to the results reported for the “Flat Queen“ variety.

It is suggested that healthy individuals should consume at least 25–30 g of dietary fibers per day [60]. Although the content of fiber in fresh fruits and vegetables is not high enough to satisfy this presumption, eating a 100 g of the investigated varieties will deliver approximately 3 g of TDF (about 10% of the minimally recommended daily intake) which could be used as an additional source of dietary fibers in the human diet. All three varieties have similar (statistically not different) amounts of TDF: around 3 g/100 g fw. The “Evmolpiya” variety had the lowest amount of SDF because of the higher IDF content. Similar results were reported for Israeli peaches (Prinus persica): 2.6–3.3 g/100 g [61]. Lower amounts of dietary fiber, were reported (without specifying the variety) by Schakel et al. [62]: around 2.0 g TDF/100 g, 1.21 g IDF/100 g and 0.80 g SDF/100 g fresh fruits. Contrary to the present results are the data for other Prunus persica L. varieties grown in Bulgaria: “Filina“, “Gergana“, “Ufo-4“, “July Lady“, and “Laskava“ [41]. The amounts of SDF in “Flat Queen“ and “Morsiani 90“ were 30–50% higher than the others, which makes them a good source of soluble fibers. Due to their pronounced gelling behavior, these soluble polysaccharides could decrease the rate of gastric emptying and influence small intestinal transit time, which explains their hypoglycemic properties and beneficial effect on human constitution [63].

Carotenoids are natural pigments that are responsible for the yellow, orange and red color of fruits, and some of them are important Vitamin A precursors [45,64]. In this context, a HPLC-DAD analysis of carotenoid content was conducted. It differed signifi-cantly among the investigated samples (Table 5). Total carotenoid content varied from 501.72 µg/100 g fw (“Flat Queen”) to 3721.97 µg/100 g fw (“Evmolpiya”). Lycopene is not usually present in many fruit and vegetables [65]. The lycopene ratio of the three varieties is 1:5.6:4 (“Flat Queen”:”Evmolpiya”:”Morsiani 90”). Its presence and higher content is proportionally linked to the red color of the fruit or vegetable, which is the case of the “Evmolpiya” variety. Lycopene’s red color is due to the many conjugated carbon double bonds, as it absorbs more visible spectrum compared to other carotenes [66].

Lutein, which is usually found in fruit and vegetables, was the dominant carotenoid in the “Flat Queen” variety (292.99 µg/100 g fw), while β-carotene (2632.27 µg/100 g fw and 1224.55 µg/100 g fw, respectively) was the most abundant in the “Evmolpiya” and “Morsiani 90” samples. The white-flesh “Flat Queen” variety possessed the lowest quantity of β-carotene and the lowest total carotenoid content in comparison to the yel-low-fleshed “Evmolpiya” and “Morsiani 90” varieties. These trends were consistent with previously observed results in different cultivars of white- and yellow-flesh peaches and nectarines varieties. The reported content of β-carotene in the yellow-flesh nectarines and peaches was about ten to thirty times higher than in the white-flesh ones [67].

Similarly, Aubert et al. [68] estimated that β-carotene is predominant and accounted for 95−97% of the total carotenoid content (26 to 197 μg/100 g) in the yellow-flesh nec-tarines harvested at commercial ripeness stage. The content of β-carotene of nectarines and peaches, reported by Vicente et al. [64], is approximately the same (150–160 μg/100 g). According to previous analyzes of the carotenoid content of early- and mid-ripening peaches and nectarines [41,45], the late-ripening varieties, contained about twice the amount of β-carotene. These findings prove the relationship between the maturity of peach fruit, the carotenoid content, and the colorimetric values of the “a” CIELAB measurement of the peach skin.

Among carotenoids, β-carotene is the main Vitamin A precursor, that is essential for the normal growth, reproduction, vision, and resistance to infection [69]. Furthermore, some investigations have suggested the preventive benefits of β-carotene against lung and colorectal cancer [70,71,72]. Total carotenoids are commonly high in yellow-fleshed peaches [73], which explains the “Evmolpiya” values. The high variability of the total carotenoid content could be justified by a variety-based diversification of carotenoid synthesis as well as it could be due to the fact, that vividly colored fruit usually contain lycopene and β-carotene as main compounds [74].

An analysis of the tocopherol content was conducted as well. Only α-tocopherol, which is the most biologically active form of vitamin E, was found (Table 5). The “Mor-siani 90” variety had the highest concentration of α-tocopherol (395.75 µg/100 g fw), fol-lowed by “Evmolpiya” (258.27 µg/100 g fw) and “Flat Queen” (245.12 µg/100 g fw). δ-tocopherol and γ-tocopherol were not present in the samples. According to Vicente et al. [64] the average content of α-tocopherol in peaches was 0.73 mg/100 g, which is about twice as high as in this study. Contrary to the results of Vicente et al. [64], the α-tocopherol content in fresh peaches, established by Durst and Weaver [75] was in the range from 1.42 ± 0.42 to 1.68 ± 0.24 mg/kg. The observed variations in the tocopherol content can be explained by the differences in the genotype of the varieties, maturity stage, environmental factors, etc.

**Figure 2:**
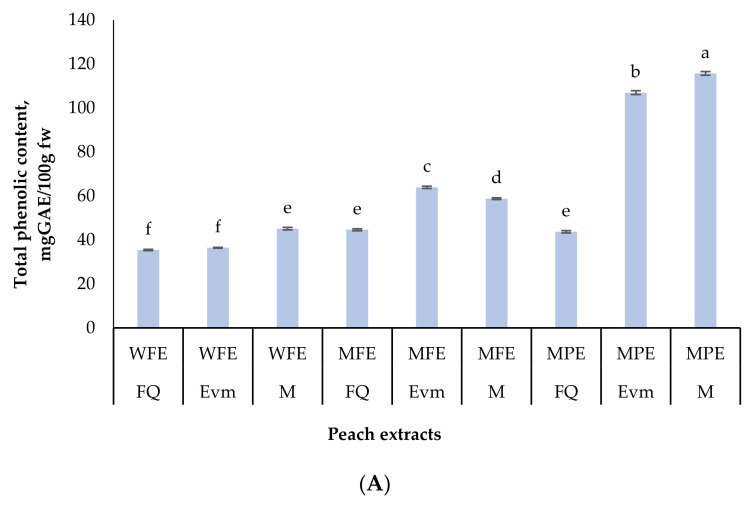

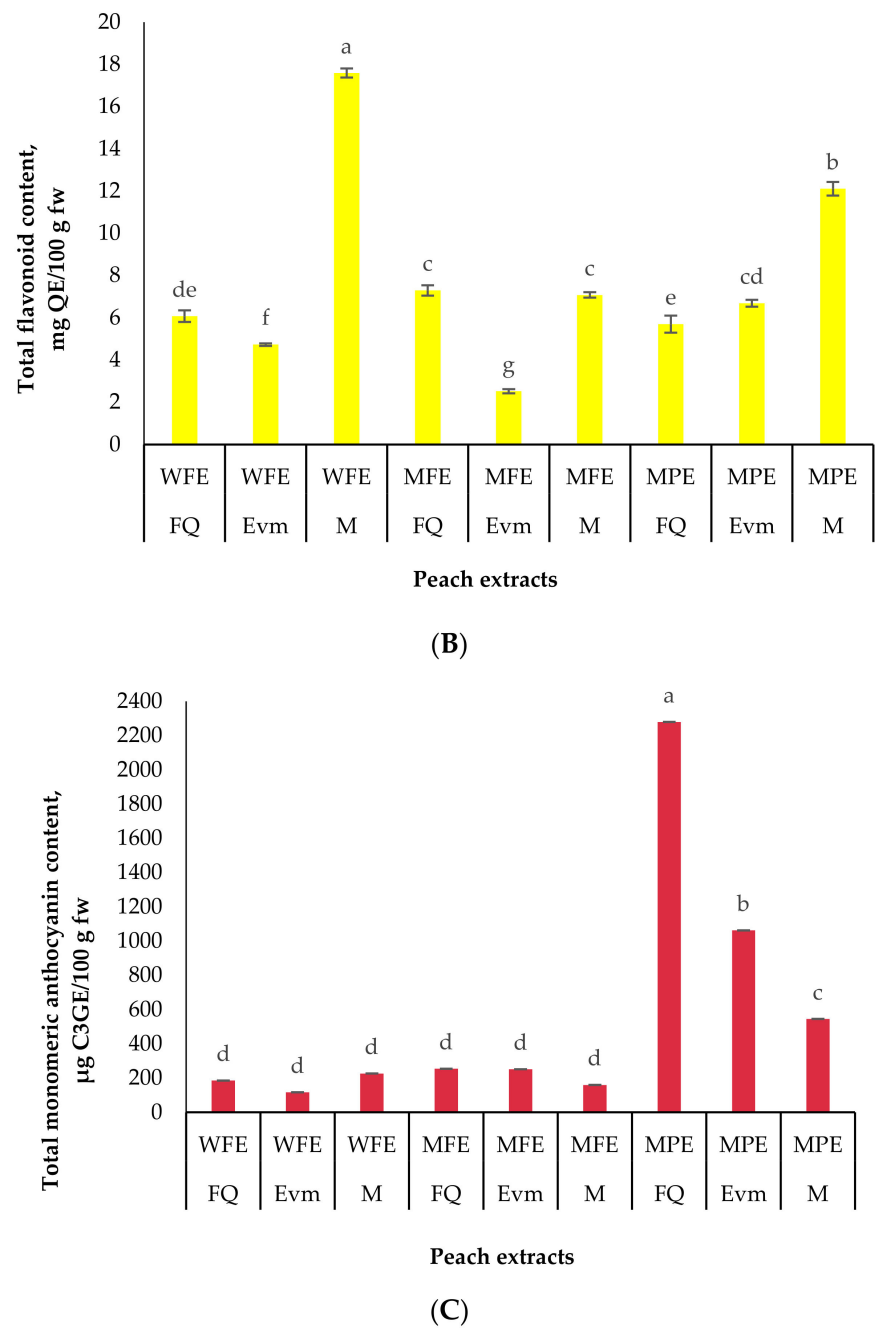
Total phenolic content (mg GAE/100 g fw) (**A**), total flavonoids (mgQE/100 g fw) (**B**) and total monomeric anthocyanins (µg cyanidin-3-glucoside (C3GE)/100 g fw) (**C**) of late season peach (*Prunus persica* L.) varieties. Values are means, *n* = 3 per treatment group. Different letters (a-g) within chart columns indicate significant differences (*p* < 0.05) between treatments as analyzed by two-way ANOVA and the Tukey test. The *p*-value for each treatment group (variety or extract), and their combination was less than 0.001. M—“Morsiani 90”; FQ—“Flat Queen”; Evm—“Evmolpiya” varieties WFE—water peach extract, MPE—methanolic peach-peel extract, MFE—methanolic peach extract.

Phenolic compounds are a diverse group of phytochemicals with complex chemical structure, degree of polymerization, and complicated interaction with other molecules. Hence, they are poorly bioavailable [76]. In in vitro assays, different solvents have been tested and vast variation of the data exist [77]. In addition, there is no single solvent that can extract all polyphenols [78]. In the present study, two type of solvents were used—water and 80% aqueous methanol. Peel and whole fruit were analyzed for total phenolic, flavonoid and anthocyanin content, some individual phenolic acids, as well as antioxidant activity. The results are presented in Figure 2. Two-way ANOVA was con-ducted in order to compare the main effects of variety and type of extract, as well as the interactive effect of both, on the total phenolic content. “Morsiani 90” and “Evmolpiya” peel extracts seemed to be the richest of TPC, resulting in 115.71, and 107.01 mg GAE/100 g fw, resp. The phenolic compounds in the other two extracts were less than 65 GAE/100 g fw. These results are comparable to the reported by Liu et al. [79] and much less to the ones by Mokrani and Madani [80]. 

Regarding the flavonoid content, “Morsiani 90” confirmed its higher concentrations. The whole fruit water extract had 17.59 mg QE/100 g fw, indicating the presence of fla-vonoid glycosides [81]. In the methanolic peel extract, 12.11 mg QE/100 g fw were re-ported. Large variations between varieties are noted by Liu et al. [79]: 17.76 ± 0.62 to 130.17 ± 0.81 mg RE/100 g fw (pulp), and 91.66 ± 1.05 to 299.86 ± 0.76 mg RE/100 g fw (peel). 

Anthocyanins are often analyzed separately, even though they are part of the fla-vonoids group. They are reported as the main contributors to the coloration of fruits, being responsible for the majority of the red to blue color shades, and patterns in fruits [82,83]. Flesh color can indicate anthocyanin content [73]. In this study, expectedly, the monomeric anthocyanins were concentrated in the peel of the fruits, resulting in 2279.33 (“Flat Queen“), 1061.67 (“Evmolpiya”), and 544.57 (“Morsiani 90”) µg C3GE/100 g fw. The content in the other extracts was under 250 µg C3GE/100 g fw, not statistically different between samples. These results are directly linked to the established CIELAB values (Table 3), where the higher “a” values indicate red predominance of the color, and the lower “b” values show a blue shade.

The individual phenolic acids of the peach extracts were also evaluated, and the results are shown in Table 6. The HPLC-DAD analysis revealed the presence of proto-catechuic, chlorogenic acid, p-coumaric, and sinapic acids. The estimated concentrations differed significantly between varieties and extracts of the same variety, as well. Chlorogenic acid was the predominant phenolic acid in all analyzed samples; the highest content was found in the peel extracts—315.10 (“Flat Queen“), 428.95 (“Evmolpiya”), and 986.95 (“Morsiani 90”) µg/g fw. This result is consistent with other studies related to the fact that chlorogenic acid is one of the main phenolic acids in different peach varieties [45,79,84,85,86,87]. This phenolic acid is associated with various valuable biological activities, such as anti-inflammatory [88], anticancer [72,89], antioxidant [90], antiepileptic, neuro-protective [91], antidiabetic [92], antimicrobial activity [93] and others. The calculated total content ranged from 70.2 to 1067.9 µg/g fw.

The abundance of chlorogenic acid, in combination of an elevated flavonoid content in “Morsiani 90” affirms that higher phenolic content is usually present in the white and yellow flesh peaches/nectarines, and is not exclusive to red-flesh fruits.

**Figure 3:**
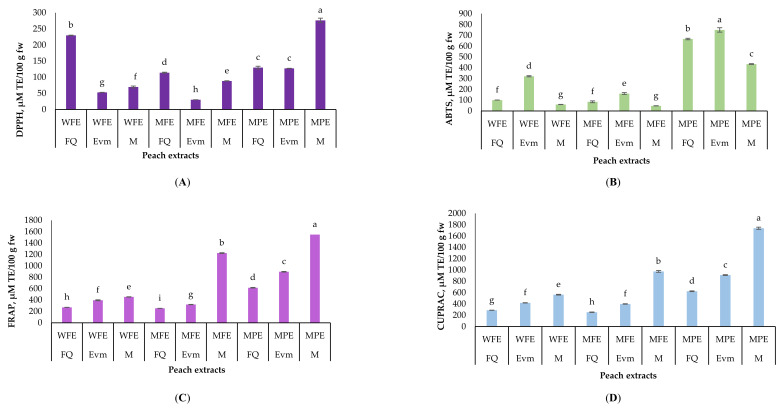
Antioxidant activity of late season peach (*Prunus persica* L.) varieties (µMTE/100 g fw) by (**A**) 2,2-diphenyl-1-picrylhydrazyl (DPPH) radical scavenging activity, (**B**) 2,2′-azino-bis(3-ethylbenzothiazoline-6-sulfonic acid (ABTS) radical scavenging activity, (**C**) ferric-reducing antioxidant power (FRAP) and (**D**) cupric ion reducing antioxidant Capacity (CUPRAC) assays. Values are means, *n* = 3 per treatment group. Different letters (a–h) within chart columns indicate significant differences (*p* < 0.05) between treatments as analyzed by two-way ANOVA and the Tukey test. The *p*-value for each treatment group (variety or extract), and their combination was less than 0.001. M—“Morsiani 90”; FQ—“Flat Queen”; Evm—“Evmolpiya” varieties. ** WFE—water peach extract, MPE—methanolic peach-peel extract, MFE—methanolic peach extract.

Evaluation of the antioxidant potential of natural resources is of critical demand in respect of the modern human’s health lifestyle context. The antioxidant activity of the studied varieties extracts was assessed by four generally recognized methods. It is im-portant to use several methods in order to get the overall antioxidant potential of any food matrix. Two-way ANOVA was conducted to study the effect of variety and extract, as well as their combination. Overall, the peel extracts were the richest in antioxidants according to all four methods (Figure 3). However, the antioxidant activity of fruits and vegetables determined by different antioxidant assays could give quite different results [94,95]. The highest Trolox equivalents per 100 g fw were detected in the peel extract of “Morsiani 90” followed by the methanolic extract of the whole fruit. Several research papers pointed a higher antioxidant activity potential of the peels compared to the cortex, mainly contributed to phenolic compounds [41,95]. According to the DPPH assay, the values varied from 30.09 to 276.28 µM/100 g fw. The peel extract of the “Morisani 90” variety possessed the highest antioxidant potential. The peel extracts had the strongest antioxidant response to the ABTS method, resulting in 433.89 (“Morsiani 90”), 665.61 (“Flat Queen“), and 749.67 (“Evmolpiya”) µM/100 g fw. Those results could be contrib-uted to the highest total monomeric anthocyanins content in the peels extracts of all three late season varieties. DPPH, FRAP and CUPRAC assays underlined that the “Morsiani 90” variety had the highest antioxidant potential.

### 2.4. Principal Component Analysis

**Figure 4:**
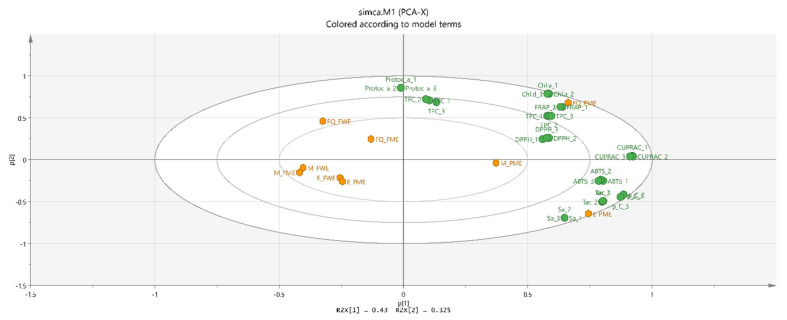
Biplot of PCA analysis of phytocomponents in late season peach (*Prunus persica* L.) varieties.M—“Morsiani 90”; FQ—“Flat Queen”; Evm—“Evmolpiya” varieties; WFE—water peach extract, MPE—methanolic peach-peel extract, MFE—methanolic peach extract; TPC_1-3—total phenolic content; TFC_1-3—total flavonoid content; Tac_1-3—total monomeric anthocyanin content; DPPH_1-3—DPPH radical scavenging assay; ABTS_1-3—radical scavenging assay; FRAP_1-3—ferric-reducing antioxidant power assay; CUPRAC_1-3—cupric ion-reducing antioxidant capacity; protoc. a_1-3—protocatechuic acid; Chl.a_1-3—chlorogenic acid; p_C_1-3—p-coumaric acid; Sa_1—sinapic acid.

In order to summarize the large set of results, and to study the interactions between analyses, principal component analysis (PCA) was conducted. PCA is the most commonly used grouping technique to determine how one sample differs from another. In the present study, 11 variables—TFC, TPC, DPPH, ABTS, FRAP, CUPRAC, total monomeric anthocyanins and each individual phenolic acid were included (Figure 4). The results showed that the two principal components explained 75.5% of the total variance. The first principal component (PC1) explained 43.0% of the total variance while PC2 explained 32.5%. PC1 interpreted the sinapic, chlorogenic, p-coumaric acids, total monomeric anthocyanins and FRAP-antioxidant activity. PC2 is worse correlated with the variables than PC1. This is to be expected, because PCs are extracted successively, each one accounting for as much of the remaining variance as possible. PC2 was mainly attributed to protocatechuic acid and total flavonoid content, which are collocated together. TPC, DPPH and FRAP antioxidant activity, as well as the chlorogenic acid data are clustered together on the right-hand side of the loading plot. When the data about the peach fruit and peach peel extracts were plotted, differences were noted. The location of the peel extracts of the varieties “Flat Queen” and “Evmolpiya” showed a clear distinction, which was evident between the peel and fruit extracts with both solvents used (Figure 4).

## 3. Materials and Methods

### 3.1. Fruit Samples

**Figure 5:**
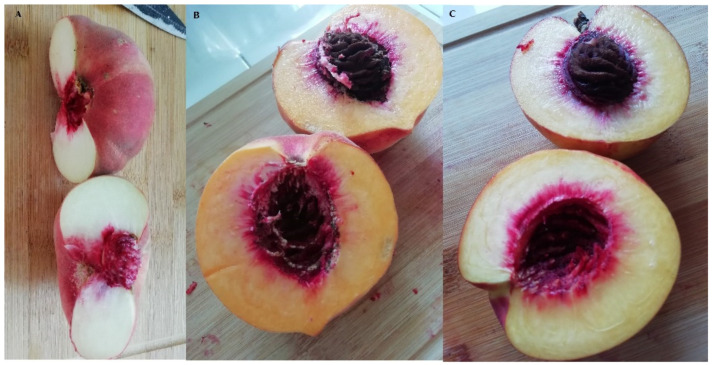
Late season peach (*Prunus persica* L.) varieties: (**A**) “Flat queen”, (**B**) “Morsiani 90” and (**C**) “Evmolpiya”.

Fruits from three late season peach varieties with different fruit characteristics were studied—“Flat Queen” (cultivar with a flat shape of the fruit), “Evmolpiya” (dessert group of peaches) and “Morsiani 90” (nectarine) grown in the same plantation. “Flat Queen” is a variety created in the USA and imported in Bulgaria in 2009 from Spain. “Evmolpiya” is a new Bulgarian variety created at the Fruit Growing Institute—Plovdiv, through interspecific hybridization with the participation of the species *Prunus persica* and *Prunus davidiana*. “Morsiani 90” is a nectarine variety created in Italy (Figure 5). 

The orchard was established in the spring of 2014. It is situated in the experimental fields of the Fruit Growing Institute—Plovdiv, Bulgaria (42°06′07.0″ N 24°43′30.7″ E). The cultivars were grafted on seed peach rootstock. The trees were trained as free-growing crowns. The plantation is grown according to standard technology for growing peaches and nectarines, according to the system “black fallow” and conventional plant protection. The orchard was not fertilized from its establishment until the time of this experiment. 

The fruits were harvested at full maturity at “ready-to-eat” ripening stage in the period from the end of August until mid-September 2020 (Table 7). 

### 3.2. Soil Sample Collection

The surface of the soil was cleared of weeds and other debris. Soil samples were collected twice in 2020 (2 March 2020 and 6 July 2020) from a depth of 0–25, 25–50 and 50–75 cm, resp. Every five sub-samples from one field were pooled and thoroughly mixed in a large plastic bucket. From this mixture, composite samples of approximately one kilo gram each were packed in plastic bags, and labelled apparently. All soil samples were dried before being transported to the laboratory, for further analysis.

### 3.3. Soil Sample Analysis

Soil samples were sieved to pass through 2 mm sieve screen, and subjected to anal-ysis of some selected soil physical and chemical parameters based on standard proce-dures as follows: soil reaction was measured by routine determination of pH using a glass electrode in a 1:5 (volume fraction) suspension of soil in water (pH in H_2_O), in 1 M/L potassium chloride solution (pH in KCl) [26].

Soil electrical conductivity was measured by HD 2206.2 conductivity meter (Delta OHM S.r.l., Padova, Italy) in aqueous extract of soil. Carbonates were measured by volumetric method [96] after the treatment of soil samples with 4 M HCl solution. Nmin (NH_4_+ NO_3_) was determined after extraction with 1 M/L KCl from soil using the Kjeldahl method by distillation unit KjelMaster K-375 (BÜCHI Labortechnik AG, Flawil, Switzerland) [97]. Mobile compounds of P_2_O_5_ and K_2_O were determined by Egner–Riem method (DL-method) [97]. Phosphorus in solution is measured calorimetrically by spec-trophotometer Halo RB-10 (Dynamica Scientific Ltd., Livingston, United Kingdom). Potassium was determined by atomic absorption spectrophotometer (AAS, Perkin Elmer 3030 B, Waltham, MA, USA). Exchangeable Ca and Mg were determined after treating the soil with 1 M NH_4_Cl solution and measurement by AAS (Perkin Elmer 3030 B, Waltham, MA, USA). The sulfate content is measured in water and acidic extracts of air-dried soils by a gravimetric method in which barium chloride is added to the water or acidic extract, and the barium sulfate precipitate is dried and weighed [98]. The total amount of micronutrients Fe, Mn, Zn and Cu as well as heavy metals Pb, Cd, Cr, Co and Ni was determined after chemical decomposition of soil with aqua regia. Cu, Zn, Mn, Pb, Cd, and Cr are measured by AAS (Perkin Elmer 3030 B, Waltham, MA, USA). Fe, Co and Ni are measured by inductively coupled plasma optical emission spectrometry (ICP-OES) instrument (Prodigy 7, Teledyne Technologies Incorporated, Hudson, USA). Humus is defined as organic carbon by sulfochromic oxidation [99]. The clay content of the soil is determined by pipette method [100].

### 3.4. Physical Attributes 

Fruit and pit were measured on a digital scale (KERN, EMB 500-1). Fruit was weight intact; afterwards the pit was extracted, and evaluated. Firmness of the fruit was meas-ured using a FT 327 fruit pressure tester (TR Turoni, Italy). Force to penetrate was ex-pressed in kg/cm^2^. Firmness was evaluated at 90, 180, and 270 degrees to the right of the suture. The color of the flesh and skin was analyzed with the use of PCE-CSM 2 (PCE-CSM instruments, Deutschland). The L*, chroma, and hue angle were evaluated. Skin color was evaluated on three locations (90, 180, and 270 degrees to the right of the suture) for each peach/nectarine. Fruit flesh was measured immediately after cutting the peach using the same technique as for the fruit skin. Total moisture content of the samples was determined according to the procedure described in AACC method 44-15.02 [101] and the ash content of the samples was determined according to the AOAC Official Method 942.05 [102]. The pH was measured using Orion 2 Star pH Benchtop (Thermo Scientific) with the electrode standardized to pH 4.0 and 7.0 Sigma buffers. Titratable acidity (TA) was measured with the use of the potentiometric method, using a pH meter Orion 2 Star pH Benchtop (Thermo Scientific). Total soluble solids (TSS) expressed as % were measured using ABBE refractometer (Carl Zeiss Abbe Laboratory Refractometer).

Maturity index (MI)—the sugar/acid ratio was calculated following the equation:

(1) $$\mathrm{MI}\;=\;\frac{\mathrm{TSS}}{\mathrm{TA}}$$

Sweetness index (SI) and total sweetness index (TSI) were calculated after HPLC analysis of individual sugars for determination of the fruit sweetness perception [103]. SI was calculated, based on the fact, that fructose and sucrose are 2.30 and 1.35 times sweeter than glucose. TSI is expressed with the contribution of each sugar estimated relative to sucrose.

### 3.5. Chemical Attributes

Peach fruit (peel and pulp) was analyzed in a fresh state for macro- and microele-ments and heavy metals by the microwave mineralization method. The analysis was performed in the accredited laboratory complex of the Agricultural University, Plovdiv. Trace elements have been determined by atomic absorption spectrometry after ash drying according to EN 14082:2003 [104]. Sodium, potassium, calcium, and magnesium contents were determined by atomic absorption spectrometry following EVS-EN 1134:2000 [105].

#### Carbohydrate, Lipid, and Fiber Analysis

The preparation of sample extracts was performed with distilled water (solid to liq-uid ratio 1:5 (*w*/*v*)) in an ultrasonic bath (VWR, Malaysia, Singapore) with a frequency of 45 kHz and 30 W power at 45 °C in triplicate. The samples were filtered. The contents of sugars and sorbitol were determined using a Shimadzu HPLC, coupled with an LC-20 AD pump, and a Shimadzu RID-10A refractive index detector (RID). The separation was done on a Shodex^®^ Sugar SP0810 (300 mm × 8.0 mm i.d.) column with Pb2+ and a Shodex SP-G guard column (5 μm, 6 mm × 50 mm) (Shodex Co., Tokyo, Japan) operating at 85 °C. The mobile phase was ultra-purified water (water purification system Adrona B30 Integrity + HPLC, Riga, Latvia) with a flow rate of 0.5 mL/min. The injection volume was 20 μL [106].

The total carbohydrate content of the samples was calculated by:

Total carbohydrates, % = 100 − (moisture, %) − (ash, %) − (protein, %) − (lipids, %)(2)

In this regard, total protein content in samples was determined by dye-binding method [107] where 100 µL of sample was mixed with 100 μL Bradford reagent (Biorad, Hercules, CA, USA). Distilled water was used as blank. The mixture was incubated at room temperature for 5 min before measuring the absorbance at 595 nm. 

The total soluble carbohydrate content was estimated by phenol-sulfuric acid method [108]. The absorbance was measured at 490 nm against blank with d. H^2^O. Total nitrogen content was determined using the Kjeldahl method according to ISO 1871 [109]. 

Total lipids content was determined by continuous extraction in a Soxhlet apparatus. Each sample (around 2 to 3 g) was packed in a pre-weighed, oven-dried thimble. The thimbles were stapled and placed in a Soxhlet apparatus, and extracted for 6 h with n-hexane. The extracts were evaporated under vacuum and the residues were weighed. The results are expressed as g/100 g fw.

The total dietary fibers were determined using a K-TDFR-100A (Megazyme, Ireland), according to AOAC method 991.43 [110] “Total, soluble and insoluble dietary fibers in foods” (First action 1991) and American association of cereal chemistry (AACC) method 32-07.01 “determination of soluble, insoluble and total dietary fibers in foods and food products” (final approval 10-16-91). Total chlorophylls were spectrophotometrically determined in 95% ethanol extracts at three wavelengths (664, 648, and 470 nm) and calculated according to Lichtenthaler and Wellburn [111]. The results are presented as g/kg fw. 

### 3.6. Extraction of Phenolic Compounds

Three extraction procedures with respect to phenolic compounds were carried out as follows:

Homogenized fresh whole fruit (peel and pulp) from each variety (each 20 g) cut into small pieces was extracted with 80% aqueous methanol (methanol:water, 80:20, *v*/*v*) at 50 °C by ultrasonication for 30 min (MEF). The residues and the extracts were separated by filtering through a filter paper; the obtained residues were re-extracted with a fresh portion of extractant in the same conditions.

The peel of fresh fruit, cut into small pieces (each 15 g), was extracted with 80% aqueous methanol (methanol:water, 80:20, *v*/*v*) at 50 °C by ultrasonication for 30 min (MEP). The residues and the extracts were separated by filtering through a filter paper; the obtained residues were re-extracted with a fresh portion of extraction solvent in the same conditions.

Homogenized fresh whole fruit (peel and pulp) from each variety (each 20 g) cut into small pieces was extracted with water by ultrasonication at 50 °C for 15 min (WEF). The extract was then subjected to heat reflux extraction for 30 min and afterwards the residues and the extracts were separated by filtering through a filter paper.

The extracts recovered from each of the extraction procedure were concentrated with a vacuum rotary evaporator (IKA RV10 digital, IKA HB 10 digital water bath -IKA^®^-Werke GmbH & Co., Breisgau, Germany) at 50 °C. The obtained semi-liquid extracts were preserved at 4 °C, until used for further experiments, but not exceeding 7 days.

### 3.7. Identification and Quantification of Phenolic Acids

The qualitative and quantitative determination of phenolic acids in the extracts was performed by using a Hitachi LaChrom Elite^®^ HPLC System (Hitachi High Technologies America, Inc., Schaumburg, IL, USA), coupled with diode-array detector (DAD, L-2455) and EZChrom Elite™ software. Separation of the phenolic acids was performed by a Supelco Discovery HS C18 column (5 μm, 25 cm × 4.6 mm), operated at 30 °C under gra-dient conditions with mobile phase consisting of 2% (*v*/*v*) acetic acid (Solvent A) and ac-etonitrile (Solvent B), as reported by Mihaylova et al. [112]. The gradient program used was: 0–1 min: 95% A and 5% B; 1–40 min: 50% A and 50% B; 40–45 min: 100% B; 46–50 min: 95% A and 5% B. The detection of phenolic acids was carried out at 280 nm for gallic, protocatechuic, and cinnamic acids and at 320 nm for chlorogenic, caffeic, ferulic, p-coumaric, sinapic, rosmarinic, and chicoric acids at a flow rate of 0.8 mL min. The re-sults are expressed in µg/g fw.

### 3.8. Carotenoid Content

Freeze-dried plant material (0.2 g) was extracted with 4 mL methanol (1:20, *w*/*v*), and then a 5 mL tetrachlormethane:methanol mixture (3:1, *v*/*v*) with 0.5% butylated hydroxytoluene added. After the extraction in an ultrasonic bath (VWR, USC200T, 60 W, 45 kHz, Magna Park Lutterworth, Leicestershire LE17 4XN, England, 60 W, 45 kHz) for 15 min, 1 mL of 10% NaCl was added. The samples were centrifuged, and the tetrachlormethane fraction was separated, filtered through anhydrous Na2SO4, and used for carotenoid analysis. Qualitative and quantitative determination of carotenoids was performed by using a LaChrom Elite (Hitachi, Tokyo, Japan) high-performance liquid chromatography (HPLC) system equipped with a diode array detector (DAD) and LaChrom Elite (Hitachi, Tokyo, Japan) software. The assay was performed according to the method described by Mihaylova et al. [113]. Separation of the carotenoids was performed on a Supelco Dis-covery HS C18 column (Sigma-Aldrich, Darmstadt, Germany, 5 μm, 25 cm × 4.6 mm), at 30 °C with a 1 mL/min flow rate of mobile phase consisting of methanol:acetonitrile (8:2, *v*/*v*, Solvent A) and tert-butyl methyl ether (MTBE, Solvent B). The gradient elution pro-gram was performed as follows: 0–0.5 min 95% Solvent A, at 3 min 80%, Solvent A, from 4.5 to 10 min 65%, A/35% B, and at 20 min 95%; A/5% B. The detection of β-carotene was carried out at 450 nm and the detection of lutein and lycopene at 470 nm. The results are expressed as μg/100 g fw, according to the established percentage of moisture content for each peach variety.

### 3.9. Tocopherol Content

Freeze-dried plant material (1 g) was saponified with a 10 mL solution (0.1 g NaCl, 4.0 g KOH, and 0.5 mg of BHT dissolved in 96% ethanol in a 50 mL volumetric flask) in a water bath at 70 °C under reflux for 30 min. After the saponification process, 15 mL 1% NaCl and a 15 mL mixture of n-hexane and ethyl acetate (9:1, *v*/*v*) were added. The organic phase was separated, vacuum evaporated to dryness, and then dissolved in 1 mL HPLC grade methanol (Sigma) for further analyses.

Separation of tocopherols was performed on a Symmetry^®^ C18 (5 μm, 15 cm × 4.6 mm) column (Waters, Milford, CT, USA) at 30 °C in isocratic mode with a mobile phase of methanol:water (98:2, *v*/*v*) with a flow rate of 2 mL/min [113]. The tocopherols were detected with a DAD at 285 nm. The results are expressed as μg/100 g fw, according to the established percentage of moisture content for each peach variety.

### 3.10. Determination of Total Polyphenolic Content (TPC)

The TPC was analyzed following the method of Kujala et al. [114] with some modifications. Each extract (0.1 mL) was mixed with 0.5 mL Folin–Ciocalteu reagent and 0.4 mL 7.5% Na_2_CO_3_. The mixture was vortexed and left for 5 min at 50 °C. After incubation, the absorbance was measured at 765 nm. The TPC is expressed as mg gallic acid equivalents (GAEs) per 100 g fw.

### 3.11. Determination of Total Flavonoid Content (TFC)

The total flavonoid content was evaluated according to the method described by Kivrak et al. [115]. An aliquot of 0.5 mL of the sample was added to 0.1 mL of 10% Al(NO_3_)_3_, 0.1 mL of 1 M CH_3_COOK, and 3.8 mL of ethanol. After incubation at room temperature for 40 min, the absorbance was measured at 415 nm. Quercetin (QE) was used as a standard and the results are expressed as mg quercetin equivalents (QE)/100 g fw.

### 3.12. Determination of Total Monomeric Anthocyanin Content

The total monomeric anthocyanin content was determined using the pH differential method [116]. Properly diluted samples were mixed with KCl (0.025 M, pH 1.0) and CH_3_COONa (0.4 M, pH 4.5) with an appropriate dilution factor. Absorbance (A) was measured using a UV–Vis spectrophotometer at 520 and 700 nm after a 15 min incubation at room temperature, and the results were calculated as follows:

A = (A_520_ − A_700_)pH 1.0 − (A_520_ − A_700_)pH 4.5 (3)

The monomeric anthocyanin (MA) pigment concentration in the samples was calculated as:

Monomeric anthocyanin pigment (mg/liter) = (A × MW × DF × 1000)/(ε × 1)(4)

where M represents the molar mass of cyanidin-3-glycoside (449.2 g/M), DF is the dilution factor, ε is molar extinction coefficient (26,900 L/M × cm), and 1 is the cuvette optical path length (10 mm). The final anthocyanin concentration is expressed as µg cyanidin-3-glucoside (C3GE)/100 g fw.

### 3.13. Determination of Antioxidant Activity

#### 3.13.1. DPPH^•^ Radical Scavenging Assay

The ability of the extracts to donate an electron and scavenge 2,2-diphenil-1-picrylhydrazyl (DPPH) radicals was determined by the slightly modified method of Brand-Williams et al. [117] as described by Mihaylova et al. [118]. A freshly prepared 4 × 10^−4^ M solution of DPPH was mixed with the samples in a ratio of 2:0.5 (*v*/*v*). The light absorption was measured at 517 nm after a 30 min incubation. The DPPH radical scavenging activity is presented as a function of the concentration of Trolox—Trolox equivalent antioxidant capacity (TEAC) and is defined as the concentration of Trolox with equivalent antioxidant activity expressed as μM TE/100 g fw.

#### 3.13.2. ABTS^•+^ Radical Scavenging Assay

The radical scavenging activity of the extracts against 2,2′-azino-bis(3-ethylbenzothiazoline-6-sulfonic acid) (ABTS^•+^) was estimated according to Re et al. [119]. Briefly, ABTS radical cation (ABTS^•+^) was produced by reacting ABTS stock solution (7 mM) with 2.45 mM potassium persulfate (final concentration) and allowing the mixture to stand in the dark at room temperature for 12–16 h before use. Afterward, the ABTS^•+^ solution was diluted with ethanol to an absorbance of 0.7 ± 0.02 at 734 nm and equilibrated at 30 °C. After the addition of 1.0 mL of diluted ABTS^•+^ solution to 0.01 mL of samples, the absorbance reading was taken at 30 °C after 6 min. The results are expressed as the TEAC value (μM TE/100 g fw).

#### 3.13.3. Ferric-Reducing Antioxidant Power (FRAP) Assay

The FRAP assay was carried out according to the procedure of Benzie and Strain [120] with slight modification. The FRAP reagent was prepared fresh daily and was warmed to 37 °C prior to use. One hundred and fifty microliters of plant extracts were allowed to react with 2850 µL of the FRAP reagent for 4 min at 37 °C, and the absorbance was recorded at 593 nm. The absorbance was recorded at 593 nm and the results are expressed as μM TE/100 g fw.

#### 3.13.4. Cupric Ion-Reducing Antioxidant Capacity (CUPRAC) Assay

The CUPRAC assay was carried out according to the procedure of Apak et al. [121]. One milliliter of CuCl_2_ solution (1.0 × 10^−2^ M) was mixed with 1 mL of neocuproine methanolic solution (7.5 × 10^−3^ M), 1 mL of CH_3_COONH_4_ buffer solution (pH 7.0), and 0.1 mL of herbal extract (sample) followed by the addition of 1 mL of water (total volume = 4.1 mL) and mixed well. Absorbance against a reagent blank was measured at 450 nm after 30 min. Trolox was used as a standard and the results are expressed as μM TE/100 g fw.

### 3.14. Statistical Analysis

Analytical determinations were performed in triplicate and the results are expressed as mean ± SD. Relevant statistical analyses of the data were performed by one-way or two-way ANOVA and a Tukey–Kramer post hoc test (α = 0.05), as described by Assaad et al. [122,123]. Principal component analysis (PCA) was applied after unit variance scaling to the data (SIMCA-P version 14.1; Umetrics, Umeå, Sweden).

## 4. Conclusions

The genus Prunus is widely spread throughout the world, with numerous varieties, possessing unique characteristics. Peaches are a well-known and strongly preferred seasonal fruit. Three commonly consumed varieties (“Flat Queen”; “Evmolpiya”; “Morsiani 90”) were studied in terms of nutritive and phytochemical content, as well as antioxidant activity. Varieties with the same flesh firmness like “Flat Queen” and “Evmolpiya” showed different pH and TA values, but similar TSS, which implies that not only the firmness, but also other chemical attributes should be taken into account to the maturity stage. The abundance of chlorogenic acid, in combination of an elevated flavonoid content in “Morsiani 90” affirms that higher phenolic content is usually present in the white and yellow flesh peaches/nectarines, and is not exclusive to red-flesh fruits. Sucrose was the major sugar in all varieties, and its value was the highest in “Flat Queen”. The total sugar value varied from 4.97 to 6.19/100 g fw.

The present study revealed that late season peach varieties (local and introduced) possess various bioactive compounds and antioxidant activity. Those, being rich in polyphenolic compounds in general, suggest a possible application in multiple areas (food processing, direct consumption, food formulations, industrial processing, and cosmetics application, etc.). The particular chemical and biological composition of the studied peach varieties can have positive effect on human health. The clarification of the composition and properties of the commercially valuable products, such as peaches, pinpoints the very important aspect, that its compositional characteristics should be available to a vast audience.

## Figures and Tables

**Table 1:** Soil properties.

Soil Parameter	Depth of the Sample					
	0–25 cm		25–50 cm		50–75 cm	
	1st *	2nd **	1st	2nd	1st	2nd
pH_(H__₂O)_	7.83 ^a^	7.52 ^d^	7.79 ^a^	7.59 ^c^	7.68 ^b^	7.64 ^bc^
pH_(KCl)_	7.06 ^b^	7.45 ^a^	7.04 ^b^	7.43 ^a^	6.81 ^c^	7.46 ^a^
EC (µS/cm)	76.30 ^e^	100.40 ^c^	65.50 ^d^	112.20 ^b^	63.90 ^d^	145.60 ^a^
Carbonates (total) (g/kg)	<1.50	<1.50	<1.50	<1.50	<1.50	<1.50
Ammonium (NH_4_-N) mg/kg)	225.84 ^b^	7.07 ^c^	214.54 ^b^	5.67 ^c^	239.52 ^a^	4.38 ^c^
Nitrate (NO_3_-N) (mg/kg)	285.12 ^a^	2.41 ^c^	<0.50	4.41 ^b^	<0.50	2.75 ^c^
N_min_ (NH_4_+ NO_3_) (mg/kg)	510.96 ^a^	9.48 ^d^	214.54 ^c^	10.08 ^d^	239.52 ^b^	7.13 ^d^
Available P (mg P_2_O_5_/100 g)	8.44 ^a^	4.47 ^c^	7.45 ^b^	2.56 ^d^	5.21 ^c^	2.75 ^d^
Available K (mg K_2_O/100 g)	32.32 ^c^	38.99 ^b^	26.91 ^d^	44.06 ^a^	21.82 ^e^	38.12 ^b^
Exchangeable Ca (mg 100/g)	94.2 ^e^	339.4 ^a^	101.2 ^d^	290.0 ^c^	103.6 ^d^	302.6 ^b^
Exchangeable Mg (mg 100/g)	39.6 ^d^	65.5 ^a^	54.7 ^b^	63.4 ^a^	45.2 ^c^	66.0 ^a^
Sulfate S (S-SO_4_. mg/kg)	546.00 ^a^	174.00 ^de^	260.00 ^b^	184.50 ^cd^	200.00 ^c^	166.38 ^e^
Iron (Fe) (mg/kg)	15,019.00 ^a^	10,396.00 ^b^	15,305.00 ^a^	15,203.00 ^a^	15,641.00 ^a^	14,783.00 ^a^
Manganese (Mn)–(mg/kg)	361.82 ^a^	320.63 ^a^	351.92 ^a^	308.72 ^a^	367.23 ^a^	298.97 ^a^
Zinc (Zn) (mg/kg)	54.72	54.00	52.36 ^a^	53.27 ^a^	43.52 ^b^	44.92 ^b^
Copper (Cu) (mg/kg)	32.63 ^ab^	26.16 ^bc^	32.30 ^a^	29.72 ^ab^	21.56 ^c^	23.31 ^c^
Lead (Pb) (mg/kg)	33.04 ^a^	15.60 ^c^	20.66 ^b^	18.72 ^bc^	16.55 ^bc^	15.58 ^c^
Chromium (Cr) (mg/kg)	15.86 ^b^	49.92 ^a^	11.90 ^b^	57.00 ^a^	15.89 ^b^	56.85 ^a^
Clay (%)	47.75		37.50		31.25	
Humus (%)	2.61		2.91		0.74	

* 1st sampling date: 02.03.2020. ** 2nd sampling date: 06.07.2020. Values are means, *n* = 3 per treatment group. Means in a row without a common superscript letter (a–e) differ (*p* < 0.05) as analyzed by one-way ANOVA and the TUKEY test.

**Table 2:** Composition attributes and quality indices of late ripening peach (*Prunus persica* L.) varieties.

Variety	“Flat Queen”	“Evmolpiya”	“Morsiani 90”
Fruit weight, g	185.63 ^b^	221.88 ^a^	193.53 ^b^
Pit weight, g	6.36 ^b^	9.86 ^a^	10.11 ^a^
Firmness, kg/cm^2^	2.33 ^b^	2.03 ^b^	5.75 ^a^
Dry matter, %	19.15 ^b^	17.69 ^b^	20.72 ^a^
Ash, %	1.16 ^a^	0.68 ^b^	1.36 ^a^
TSS, %	14.44 ^b^	14.50 ^a^	13.54 ^c^
TA	0.45 ^c^	0.65 ^a^	0.58 ^b^
pH	4.56 ^a^	3.65 ^b^	3.51 ^c^
Maturity index	32.09	22.31	23.34

TSS—total soluble solids, TA—titratable acidity, expressed as malic acid. Values are means, *n* = 3 per treatment group. Means in a row without a common superscript letter (a–c) differ (*p* < 0.05) as analyzed by one-way ANOVA and the TUKEY test.

**Table 3:** CIELAB skin and flesh color measurements of late ripening peach (*Prunus persica* L.) varieties.

Attribute/Variety		“Flat Queen”	“Evmolpiya”	“Morsiani 90”
Skin	L	42.63 ^a^	51.35 ^a^	41.35 ^a^
	a	27.47 ^a^	31.65 ^a^	30.92 ^a^
	b	18.81 ^b^	31.86 ^a^	24.01 ^ab^
	hue	32.64 ^a^	43.39 ^a^	37.53 ^a^
	chroma	33.15 ^b^	46.32 ^a^	24.01 ^c^
Flesh	L	73.36 ^a^	71.52 ^a^	71.07 ^a^
	a	1.58 ^b^	13.74 ^a^	9.27 ^a^
	b	16.65 ^b^	47.41 ^a^	43.52 ^a^
	hue	83.67 ^a^	72.81 ^b^	77.98 ^ab^
	chroma	16.92 ^c^	49.99 ^a^	44.66 ^b^

Values are means, *n* = 9 per treatment group. Means in a row without a common superscript letter (a–c, ab) differ (*p* < 0.05) as analyzed by one-way ANOVA and the TUKEY test.

**Table 4:** Mineral content of different varieties of late season peach (*Prunus persica* L.) varieties, mg/kg fw *.

Mineral Contents	Peach Varieties		
	“Flat Queen”	“Evmolpiya”	“Morsiani 90”
K	353.37	738.48	1978.34
P	300.00	200.00	300.00
Ca	< 0.13	< 0.13	< 0.13
Mg	4.11	2.77	11.84
Na	24.57	38.21	55.92
Fe	0.47	0.62	0.44
Zn	0.32	0.55	1.18
Mn	0.19	0.12	0.22
Cu	0.06	0.22	0.89
Pb	< 0.10	< 0.10	< 0.10
Cr	< 0.10	< 0.10	< 0.10
Total	683.42	981.30	2349.03
N *	9.80	1.0	4.10

* Nitrogen content is expressed as g/kg fw.

**Table 5:** Carbohydrates (g/100 g fw), total lipids (g/100 g fw), energy value (kcal), dietary fiber (g/100 g fw), carotenoids (µg/100 g fw), and tocopherols (µg/100 g fw) of late season peach (*Prunus persica* L.) varieties.

Variety	“Flat Queen“	“Evmolpiya”	“Morsiani 90”
Sucrose	4.21 ^a^	3.64 ^b^	3.41 ^b^
Glucose	0.81 ^c^	1.60 ^a^	1.32 ^b^
Fructose	0.45 ^b^	0.95 ^a^	0.24 ^c^
Sorbitol	0.37 ^b^	0.09 ^c^	0.47 ^a^
Sucrose/Glucose	5.20	2.28	2.58
Glucose/Fructose	1.80	1.80	1.80
Total sugars	5.47 ^b^	6.19 ^a^	4.97 ^c^
Total carbohydrates	11.41 ^b^	15.78 ^a^	16.21 ^a^
Sweetness index	75.3	87.0	64.8
Total sweetness index	55.0	62.8	47.7
Total lipids	0.46 ^b^	0.61 ^a^	0.59 ^a^
Energy value	74.26	71.09	80.39
Fibers			
TDF	2.92 ^a^	3.23 ^a^	3.49 ^a^
IDF	1.74 ^c^	2.54 ^a^	2.27 ^b^
SDF	1.13 ^a^	0.69 ^b^	1.17 ^a^
Carotenoids			
Lutein	292.99 ^b^	206.97 ^c^	511.78 ^a^
Lycopene	157.03 ^c^	882.73 ^a^	638.18 ^b^
β-carotene	51.70 ^c^	2632.27 ^a^	1224.55 ^b^
Total carotenoids	501.72 ^c^	3721.97 ^a^	2374.51 ^b^
Tocopherols			
δ- tocopherol	nd	nd	nd
γ- tocopherol	nd	nd	nd
α- tocopherol	245.12 ^c^	258.27 ^b^	395.75 ^a^

nd—not detected. Values are means, *n* = 3 per treatment group. Means in a row without a common superscript letter (a–c) differ (*p* < 0.05) as analyzed by one-way ANOVA and the TUKEY test.

**Table 6:** Phenolic acid content * (µg/g fw) of late season peach (*Prunus persica* L.) varieties.

Variety/Compound	Type of Extract **	“Flat Queen“	“Evmolpiya”	“Morsiani 90”
Protocatechuic acid	WFE	9.81 ^g^	34.05 ^c^	94.40 ^a^
	MPE	6.64 ^h^	21.56 ^e^	69.69 ^b^
	MFE	3.12 ^i^	19.96 ^f^	32.74 ^d^
Chlorogenic acid	WFE	83.41 ^h^	214.70 ^f^	487.32 ^c^
	MPE	315.10 ^e^	428.95 ^d^	986.95 ^a^
	MFE	56.55 ^i^	184.69 ^g^	528.92 ^b^
p-Coumaric acid	WFE	5.42 ^d^	2.41 ^fg^	2.22 ^g^
	MPE	17.83 ^a^	8.22 ^c^	8.73 ^b^
	MFE	4.53 ^e^	2.52 ^f^	2.61 ^f^
Sinapic acid	WFE	4.82 ^d^	1.10 ^h^	1.21 ^g^
	MPE	16.75 ^a^	5.50 ^c^	2.71 ^e^
	MFE	6.11 ^b^	1.31 ^f^	1.2 ^g^
Total phenolic acids ***	WFE	103.46	252.26	585.15
	MPE	356.32	464.23	1068.08
	MFE	70.31	208.48	565.4

The data are presented as the mean (*n* = 3). Different superscript letters (a–i) within each row indicate significant differences between treatments according to Tukey’s test at *p* < 0.05. * Gallic acid, caffeic acid, ferulic acid, rosmarinic acid, cichoric acid, and cinnamic acid were not detected in any extract. ** WFE—water peach extract, MPE—methanolic peach-peel extract, MFE—methanolic peach extract. *** Total–sum of the mean of individual elements.

**Table 7:** Fruit type, flesh color, harvest date, and harvest time in days after full bloom (DAFB) of the varieties. P—peach, N-nectarine, FP—flat peach, Y—yellow, W—white.

Variety	Type	Flesh Color	Harvest Date	Harvest Time (DAFB)
**“Flat Queen”**	FP	W	17 August	144
**“Evmolpiya”**	P	Y	11 September	164
**“Morsiani 90”**	N	Y	18 September	178

## Data Availability

The data presented in this study are available on request from the corresponding author.

## Sample Availability

Samples of the compounds are available from the authors.
